# *In situ* growth of halide perovskite crystals and thin films on optical fiber end facets

**DOI:** 10.1039/d5ra07875j

**Published:** 2026-01-19

**Authors:** Yang Yu, Kanak Kanti Bhowmik, Ruan Li, Kexin Li, Lin Zhu, Hai Xiao, Lianfeng Zhao

**Affiliations:** a Holcombe Department of Electrical and Computer Engineering, Clemson University Clemson South Carolina 29634 USA Lianfez@clemson.edu

## Abstract

Halide perovskites exhibit significant advantages for active optical components such as light emitting diodes, solar cells and photodetectors due to their excellent optoelectronic properties. Their nonlinear optical effects and other characteristics also make them suitable for integration into waveguide components, such as optical fibers, for applications like optical modulation. Although some efforts have been made to integrate perovskite nanomaterials with optical fibers, technological challenges have hindered reliable *in situ* preparation methods. Herein, we propose an area-selective wetting strategy for optical fibers, which utilizes hydrophobic sidewalls and hydrophilic end facets to reliably hold small precursor droplets. By introducing a space confinement strategy to suppress the kinetics of solvent evaporation, methylammonium lead bromide (MAPbBr_3_) perovskite crystals were successfully grown *in situ* on the fiber end facet. The versatility of this *in situ* growth method for perovskite crystals on fiber end facets of various sizes has also been verified. In a separate approach, the controllable *in situ* preparation of CsPbBr_3_ polycrystalline thin films was achieved through vacuum-assisted rapid crystallization. Our strategy provides a controllable platform for the integration of perovskite materials and optical fibers, enabling further development in optical applications.

## Abstract 2

An area-selective wetting strategy establishes hydrophobic sidewalls and hydrophilic end facets on optical fibers, which enables the controllable *in situ* growth of halide perovskite crystals and polycrystalline thin films on fiber end facets.

## Graphical abstract

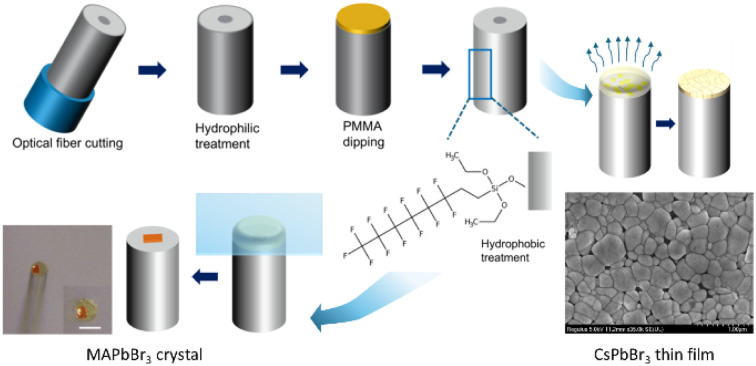

## 1. Introduction

Halide perovskites are an emerging class of optoelectronic semiconductors. They possess a compelling set of optoelectronic properties, such as a high absorption coefficient,^1^ high photoluminescence quantum yield,^2^ long carrier diffusion length,^3^ and extended radiative recombination lifetime.^4^ A key advantage is their compositionally tunable electronic band structure, which allows for precise engineering of their optical and electrical characteristics.^5^ These properties make them promising candidates for applications such as solar cells,^6^ photodetectors,^7^ light-emitting diodes (LEDs),^8^ lasers,^9^ and neuromorphic devices.^10^

It is noteworthy that beyond photovoltaics, the most significant achievements of halide perovskites in optical applications have been in active optical components like LEDs, and photodetectors. For example, Liao *et al.*^11^ reported a photonic neural network based on a hetero-integrated platform of perovskites and Si_3_N_4_, where the perovskite served as the active layer for both light emission and detection, while Si_3_N_4_ acted as the waveguide. However, passive optical components made from halide perovskites, such as filters, couplers, and splitters, have been less explored. While bottom-up, template-assisted growth has demonstrated polariton propagation in waveguides based on CsPbBr_3_ nanowires,^12^ this approach faces certain limitations. Issues with process compatibility, a lack of universal methods, and constraints on effective refractive indices make heterogeneous integration of perovskites with other optical components a more prevalent strategy.

Optical fiber, as one of today's most important optical components, is widely used in fields such as communication, sensing, and healthcare due to its wide bandwidth, low loss, and resistance to electromagnetic interference.^13^ Perovskites, with their strong absorption, efficient electro-optical and photoelectric conversion, and nonlinear effects, are complementary materials for integration into fiber-based systems for detection, emission, modulation, and processing.^14^ The solution-processability and compositional diversity of perovskites provide a feasible pathway for realizing integrated perovskite/optical fiber applications. Previous work by Li *et al.*^15^ utilized a dry-transfer method to integrate a methylammonium lead iodide (MAPbI_3_) single nanosheet onto a fiber end facet, creating a saturable absorber (SA) that achieved stable picosecond-duration soliton mode-locked laser pulses. Similarly, Jiang *et al.*^16^ reported an SA based on MAPbI_3_ with a strong non-linear response, realizing ultrafast (661 fs) mode-locked laser pulses. Inorganic CsPbBr_3_ nanocrystals have also been synthesized and drop-cast onto the end face of Er-doped fiber to create a stable dissipative soliton fiber laser.^17^ While these methods are effective, they often require pre-synthesis of the material followed by a separate transfer step, which can introduce contamination or mechanical damage. Integration based on side-polished fibers, where a precursor solution is spin-coated directly onto the polished surface, is comparatively straightforward but alters the fiber's cylindrical geometry.^18^ The potential of combining perovskite properties with optical fibers for various applications such as medical and industrial sensors,^19^ is not yet fully realized due to the limited integration methods currently available.

Most existing work on the optical applications of perovskite-fiber integration utilizes the nonlinear absorption effect of the perovskite, a property that does not require complete controllability or high-quality crystalline coverage. In contrast, using the perovskite as a gain medium for applications like fiber-integrated lasers poses a greater challenge, as it requires the deposition of high-quality crystalline material directly on the fiber end. In parallel, surface wettability engineering has emerged as a crucial approach for improving the quality of perovskite materials and enabling functional patterning. Tailoring substrate surface energy can effectively regulate precursor spreading and suppress dewetting, which is essential for fabricating high-performance solar cells and LEDs with high-quality films.^20^ Furthermore, differential wettability templates have been successfully employed to guide the growth of perovskite micro-arrays and individual crystals,^7^ paving the way for advanced optoelectronic photodetector arrays with minimized pixel sizes and high spatial resolution.^21^ Herein, we propose a systematic strategy to realize fully controlled *in situ* growth of halide perovskites on fiber ends. This idea originated from selectively controlling the wettability of different parts of the optical fiber surface to confine the perovskite precursor solution to the desired end facet.^22^ A designed treatment process results in hydrophobic sidewalls and hydrophilic end facets. Building on this droplet fixation, we designed specific crystallization strategies based on the properties of different perovskite compositions. For MAPbBr_3_, dimethyl sulfoxide (DMSO), a co-solvent with a high boiling point and low evaporation rate, was introduced, and the evaporation kinetics were further controlled using a space confinement method. This approach successfully suppressed the nucleation rate to achieve crystal growth at the fiber end. On the other hand, for all-inorganic CsPbBr_3_, which exhibits low solubility, we achieved high-quality polycrystalline thin films using a vacuum-assisted crystallization strategy. All prepared perovskites exhibited strong and compositionally correct photoluminescence peaks, confirming their quality and indicating their potential for further application as optical gain media integrated on fiber ends.

## 2. Results and discussion

**Fig. 1:**
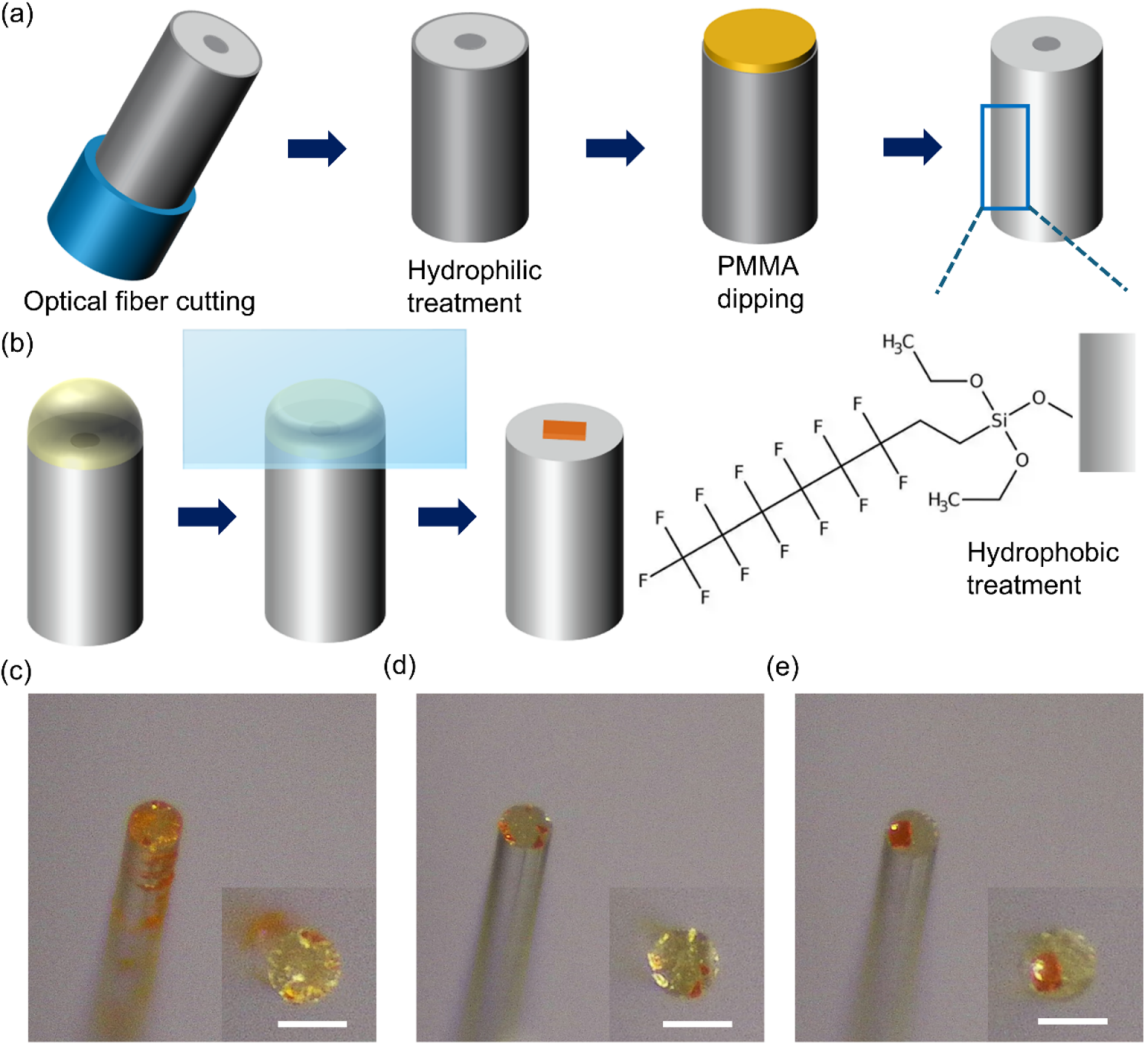
Strategy of hydrophobic sidewall and hydrophilic end facet for growth of MAPbBr_3_ individual crystals. (a) Schematic diagrams of process procedures. (b) Schematic diagrams of space confinement method for crystal growth on fiber end surface. Optical images of MAPbBr_3_ crystals on optical fiber with (c) fully hydrophilic surface, (d) fully hydrophobic surface and (e) hydrophilic end and hydrophobic sidewalls. Insets in (c–e) are the top view photos. Scale bar is 125 µm.

One challenge in growing halide perovskite on a fiber end is confining the precursor solution to the end facet. We have designed a feasible process to confine solution droplets to the end face and prevent their migration to other areas, such as the fiber sidewall. The entire process is shown in Fig. 1a. It aims to make the fiber sidewall hydrophobic while leaving the fiber end hydrophilic. First, the optical fiber is stripped of its outer acrylate coating and cleaved by a diamond blade to obtain an exposed, flat end. Then, the fiber is treated with UV light ozone or oxygen plasma to make the entire silica surface clean and hydrophilic. Subsequently, the goal is to protect the end facet, which has already undergone hydrophilic treatment, so that it is unaffected by the following hydrophobic treatment for the sidewall. Although there are many mature methods for depositing materials on substrates, selectively covering only the fiber end face remains challenging. We developed a method combining dipping and spin-coating operations to selectively apply a protective layer to the fiber end surface. A layer of PMMA solution is spin-coated on a hydrophilic glass surface; the optical fiber is then held vertically and brought into contact with the still-wet liquid film. In this way, only the end surface of the optical fiber is coated with PMMA. For this process, a modified PMMA solution was used; introducing dimethylformamide (DMF), a solvent that evaporates slowly, into toluene and reducing the spin-coating time yields an uncured PMMA film that remains workable for the dipping step. After that, the fiber is placed inside a chamber with trichloro(1*H*,1*H*,2*H*,2*H*-perfluorooctyl)silane under vacuum. In this vapor-phase deposition environment, the functional silane molecules evaporate and distribute throughout the chamber. The molecule's reactive Si–Cl groups bond to the surface hydroxyl groups on the silica fiber sidewall, forming a durable self-assembled monolayer with the hydrophobic fluorinated alkyl group facing outward. The final procedure is to soak the samples in an organic solvent to dissolve and remove the protective PMMA on the fiber end. With this process, the resultant silica optical fibers can hold polarized solutions on the hydrophilic end surface and prevent these solutions from spontaneously flowing down the hydrophobic sidewalls.

To demonstrate the wettability changes more clearly, side-view photographs of water droplets on the five different surfaces are presented in Fig. S1. These images provide intuitive visual evidence of the surface energy transitions: (a) bare glass exhibits a moderate hydrophilic state; (b) UV ozone-treated glass shows almost complete spreading, indicating a super-hydrophilic state due to the removal of organic contaminants and the generation of hydroxyl groups; (c) PMMA-coated glass recovers a higher profile, reflecting the polymer's intrinsic hydrophobicity; (d) PMMA-removed glass returns to a wetting behavior similar to the UV ozone-treated glass, confirming the restoration of hydrophilicity; and (e) hydrophobic treatment results in a nearly spherical droplet with a significantly high contact angle, demonstrating a successful conversion to a hydrophobic state. These side-view observations clearly validate the wettability tuning throughout our fabrication process. The PMMA layer acts as a sacrificial protective mask in this process. Importantly, no heating was used to sinter the PMMA; this uncrosslinked state facilitates its subsequent dissolution by organic solvents. The solvents used for removing PMMA (*e.g.*, DMF, toluene, or chlorobenzene) are chosen specifically because they effectively dissolve PMMA polymer chains but are chemically inert toward the siloxane (Si–O–Si) bonds that constitute the hydrophobic functional layer on the sidewalls. As shown in Fig. S1e, the hydrophobic treatment maintains a very high contact angle even after these cleaning protocols.

Merely fixing the solution to the end face of the optical fiber is insufficient for growing a perovskite crystal. Such small-sized droplets will evaporate quickly, and the limited solute will rapidly nucleate, forming multiple dispersed small crystals simultaneously. To address this, we referred to the idea of space confinement, a technique commonly used in growing single-crystal thin films.^9,22^ This involves covering the solution droplet on the fiber end with a hydrophobic glass slide to reduce the area of the liquid–gas interface and significantly decrease the volatilization rate, thereby achieving reliable growth of perovskite crystals on the end face, as shown in Fig. 1b.

To verify the effectiveness of the area-selective hydrophilic/hydrophobic treatment strategy, we prepared optical fibers with fully hydrophilic, fully hydrophobic, and our target configuration (hydrophilic end face and hydrophobic sidewall). We dropped a 0.9 M MAPbBr_3_ solution (solvent ratio of DMF : DMSO is 1 : 4) onto the end facets and allowed them to grow naturally at room temperature under the confinement of a hydrophobic glass slide. Fig. 1c–e show optical microscope images of the three sets of samples from a tilted perspective. A fully hydrophilic optical fiber makes it difficult to control the solution, which tends to be squeezed down the sidewall when the glass is applied. The subsequent evaporation of the solvent leads to the formation of crystals in the area where the solution has spread. For fully hydrophobic optical fibers, crystallization on the sidewall does not occur, but it is also impossible to grow a crystal at the end. Heterogeneous nucleation of crystals depends on surface energy, and both the hydrophobic end face and the glass cover are thermodynamically unfavorable for the formation of crystal nuclei. Kinetically, the liquid–air interface at the droplet's edge is where the solvent evaporates outward most rapidly, allowing the solution there to reach the supersaturation required for crystallization first. This explains why crystals tend to distribute around the outer circular area of the hydrophobic fiber end. Finally, an individual crystal was successfully obtained for the sample with a hydrophilic end face and hydrophobic sidewalls. The hydrophilic end face provides an advantageous nucleation site, the hydrophobic sidewall ensures the solution droplet is confined to the end face, and the hydrophobic glass cover effectively suppresses the rapid evaporation of the solvent. This area-selective wetting strategy successfully demonstrated the ability for controllable growth of a MAPbBr_3_ crystal on the fiber end.

MAPbBr_3_ was selected as the model compound for this study because it is a prototypical halide perovskite with a stable crystal structure and an ideal structural tolerance factor. Its fundamental properties, such as lattice constant and bandgap, are intermediate to those of its chlorine- and iodine-based counterparts.^23,24^ Crucially, MAPbBr_3_ is particularly well-suited for demonstrating crystal growth. It exhibits moderate solubility in common organic solvents like DMF and DMSO and possesses an inverse temperature solubility curve that is advantageous for crystallization. These characteristics permit the use of multiple straightforward fabrication techniques, including slow solvent evaporation,^25^ inverse temperature crystallization,^26^ and anti-solvent induced crystallization.^27^ In contrast, MAPbI_3_ requires high-temperature processing with γ-butyrolactone (GBL),^28^ and forms a different, albeit photoactive, tetragonal phase near room temperature, adding experimental complexity.^29^ Therefore, the accessible and reliable preparation of MAPbBr_3_ makes it the ideal candidate for exploring *in situ* crystal growth on optical fiber end facets.

**Fig. 2:**
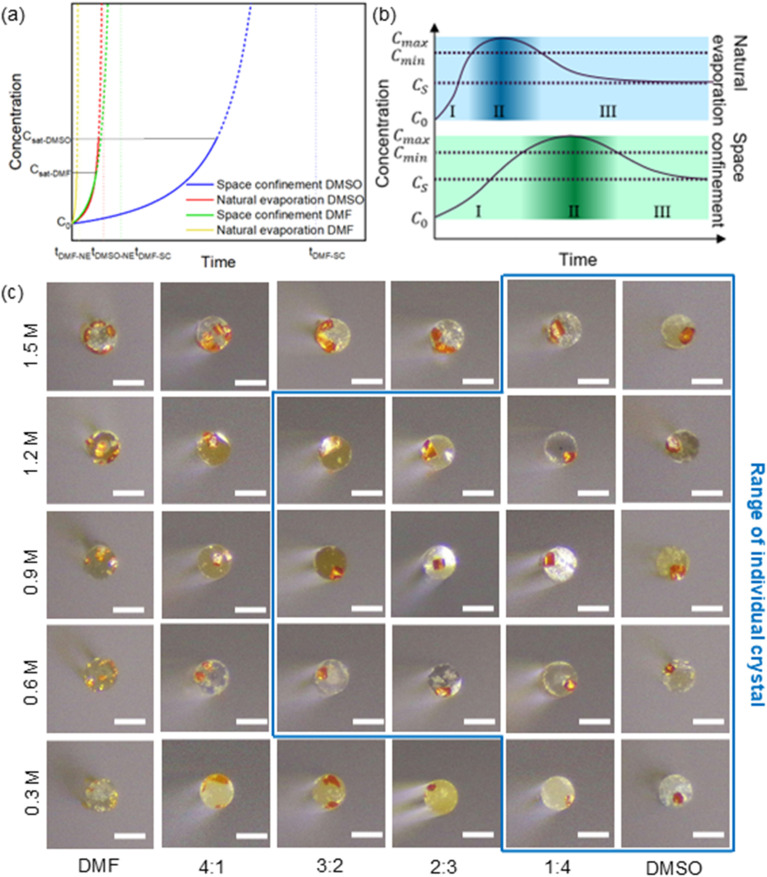
Controlled growth of MAPbBr_3_ perovskite individual crystals on fiber end. (a) Schematic representation of the relationship between evaporation time and concentration of the solutions under different conditions and various types of solvents. (b) Predicted crystallization stages based on LaMer model for conditions of space confinement and natural evaporation. (c) Optical images of crystallization results for different ratios of DMF and DMSO solvents and concentrations ranging from 0.3 M to 1.5 M. Scale bar is 125 µm.

To further understand the crystallization kinetics of this area-selective wetting strategy, we further studied the effects of solvent composition and solute concentrations. We started from the principle of crystallization and analyzed the evaporation kinetics under different conditions, solvents, and concentrations, specifically focusing on the relationship between solution concentration and time. The derivation of a simplified model can be found in the SI (Note 1). As shown in Fig. 2a, there is a significant difference in the concentration change curves between samples with spatial confinement and those without. Without the coverage of the hydrophobic glass, the solution droplets are fully exposed, and the solvent evaporates rapidly from the solution–air interface. Under the effect of space confinement, the concentration of the solution changes very slowly, then increases rapidly near the end of the evaporation process. It can be approximately estimated that the confinement condition delays the volatilization process by several times.

Solvent selection is another decisive factor. Different solvents have different evaporation rates; the evaporation rate for DMF is at least several times higher than that of DMSO. Another effect of solvents on crystallization is the solubility of the solutes. Although the degree of supersaturation directly determines the crystallization process, the maximum soluble concentration can be used to indirectly estimate its timing. The solubility of MAPbBr_3_ in DMF is less than 2 M, whereas DMSO can dissolve up to 3 M. Therefore, based on volatility and solubility, under the same conditions and initial concentration, the crystallization of MAPbBr_3_ in DMF will begin significantly earlier than in DMSO.

While our model explored concentration changes by assuming infinite solubility, real-world solutions have limited solubility. Consequently, as the solvent evaporates and the solution concentration reaches a critical point, a phase transition occurs, causing crystals to precipitate. This process of nucleation and growth is well-described by the LaMer model.^30^ The crystallization process of a solution normally goes through three stages: stage one, where the concentration gradually increases as the solvent evaporates; stage two, where crystal nuclei form once a critical supersaturation level is reached; and stage three, where existing crystals grow larger as more solute precipitates. As mentioned earlier, space confinement and fully exposed natural volatilization led to completely different evaporation rate curves. As shown in Fig. 2b, as the solution evaporates, its concentration increases from an initial *C*_0_ to the nucleation point, *C*_min_. The concentration peaks at *C*_max_ before decreasing due to solute depletion from crystal growth. Under the condition of space confinement, the significantly reduced evaporation rate greatly delays the beginning of stage two. More importantly, the duration of this nucleation stage also increases, which means that the nucleation rate is greatly decreased, providing the opportunity for the formation of individual crystals. After the end of stage two, the solution will remain at its saturation concentration, and the continuous evaporation of the solvent will induce the existing crystals to grow larger rather than forming new nuclei.

Fig. 2c shows the growth of MAPbBr_3_ crystals under spatially limited conditions using solutions with different concentrations (from 0.3 M to 1.5 M) and solvent compositions (pure DMF, pure DMSO, and mixtures). The composition of the solvent is the decisive factor in determining whether an individual crystal will form, regardless of the solution concentration. In high-boiling-point solvents (pure DMSO or DMSO-rich mixtures), there is a strong tendency to grow a individual crystal. In this case, the solution concentration primarily determines the final size of the crystal, as the amount of solution held on the fiber end face is roughly equal for a given contact angle. Furthermore, the differences in wettability among solution droplets composed of different solvent ratios on surfaces treated with the same process are negligible, as shown in Fig. S3. For DMF-rich or pure DMF solutions, their rapid evaporation and narrow nucleation window make it almost impossible for the solution to grow individual crystals under these conditions. This experimental observation is consistent with the conclusion derived from the evaporation model in the SI (Note 1).

**Fig. 3:**
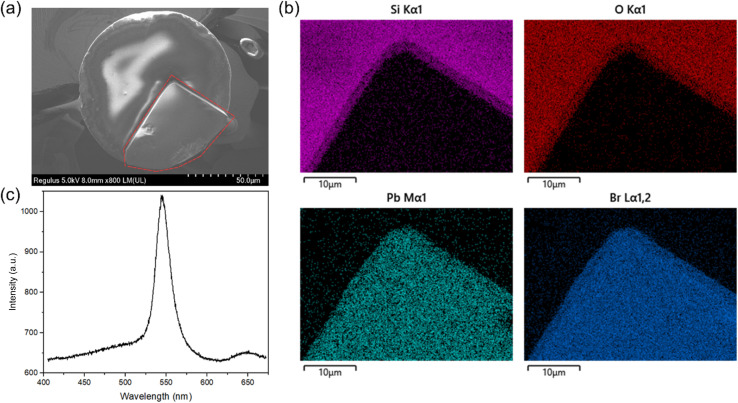
Material characterization of MAPbBr_3_ individual crystal on fiber end. (a) SEM image, where red dashed lines show the boundaries of the perovskite crystal, (b) EDS mappings and (c) PL spectrum of the MAPbBr_3_ crystal.

The material composition of the grown MAPbBr_3_ was further characterized by scanning electron microscopy (SEM) and photoluminescence spectroscopy. The SEM image (Fig. 3a) shows the complete crystal morphology of the MAPbBr_3_ crystal, where red dashed lines indicate the boundaries of the perovskite crystal. Although the circular fiber end face limits its growth dynamics, preventing the formation of a perfect cube, its surface is smooth and continuous, showing no evidence of grain boundaries. The energy-dispersive X-ray spectroscopy (EDS) mapping in Fig. 3b demonstrates the correct, strong, and uniform distribution of the characteristic elements Pb and Br, while the exposed surface of the optical fiber only contains signals of Si and O. Finally, its photoluminescence curve, shown in Fig. 3c, exhibited a peak at around 535 nm, consistent with previous literature reports,^9^ thereby verifying the composition of the MAPbBr_3_ crystal prepared at the fiber end facet. In contrast, the photoluminescence spectrum of a bare optical fiber sample without perovskite integration showed no fluorescence peaks (Fig. S2), effectively demonstrating that the signal in the integrated sample originated solely from the photoactive perovskite component.

**Fig. 4:**
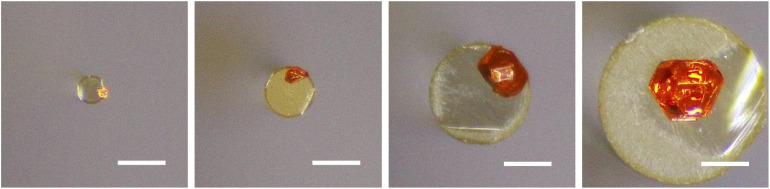
Optical photos of MAPbBr_3_ individual crystals grown on visible light optical fibers with diameters of 105, 200, 400 and 600 µm (from left to right, scale bar: 200 µm).

The optical fibers we studied so far have a diameter of 125 µm. To verify the reliability of our method for preparing perovskite on fiber end facets with different diameters, we used 105, 200, 400, and 600 µm multimode visible light fibers for validation. As shown in Fig. 4, MAPbBr_3_ crystals can be grown on all the above-mentioned sizes of optical fibers, which paves the way for the subsequent integration of perovskite-based optics with a wider range of optical fibers.

**Fig. 5:**
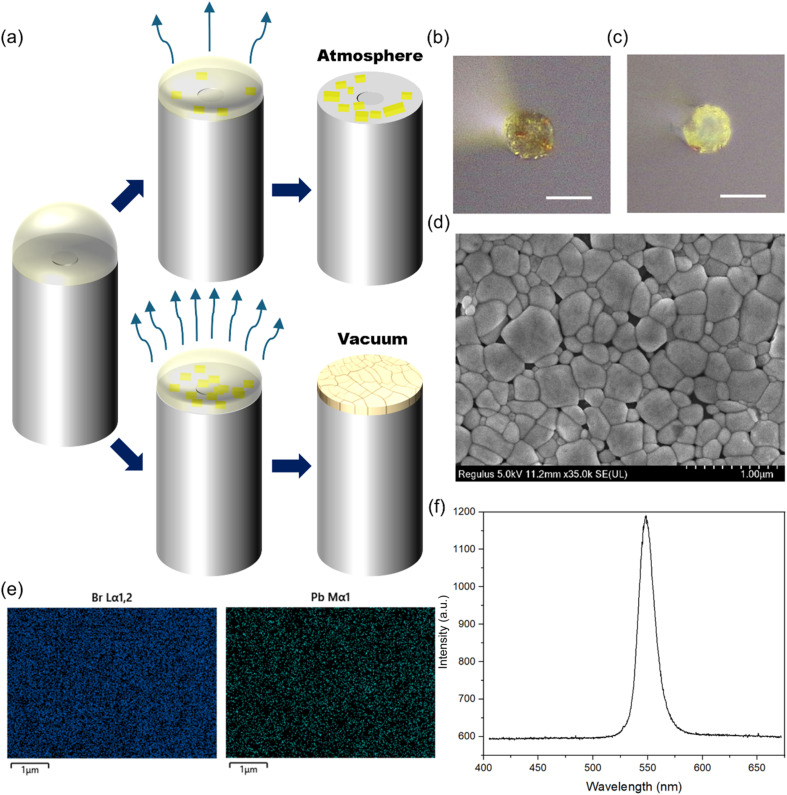
Controllable growth of CsPbBr_3_ thin film on optical fiber end under vacuum condition and its material characterization. (a) Schematic diagrams of the principle of the vacuum assisted growth method. Optical images of (b) sample grown by naturally evaporated solution and (c) vacuum assisted growth of CsPbBr_3_ thin film on fiber ends. (d) SEM image of the as-prepared CsPbBr_3_ thin film on fiber. (e) EDS mapping of elements Br and Pb of the corresponding area of CsPbBr_3_ thin film in (d). (f) PL spectrum of the CsPbBr_3_ thin film on fiber end.

While controlled solvent evaporation is effective for growing perovskite individual crystals, this method makes it challenging to achieve full, continuous coverage on the fiber end facet. For applications that require a complete layer, we therefore shifted our focus to fabricating high-quality polycrystalline thin films using a different approach. The formation of such films depends on inducing high-density nucleation across the substrate in a very short time. To explore this, we used the all-inorganic perovskite CsPbBr_3_. Fig. 5a provides a mechanistic comparison of CsPbBr_3_ film formation. The upper pathway represents growth under atmospheric pressure, where slow solvent removal results in localized nucleation and a fragmented morphology. Conversely, the lower pathway depicts the vacuum-assisted strategy. By placing the entire fiber facet into a vacuum chamber, the rapid extraction of solvent induces a burst of nucleation. This high nucleation density effectively suppresses the growth of isolated large grains, instead promoting the formation of a seamless and uniform polycrystalline thin film covering the entire end facet. All-inorganic perovskite precursors generally exhibit low solubility in polar organic solvents. Consequently, a saturated solution of these precursors is highly sensitive to changes in physical conditions, readily becoming supersaturated and promoting a high nucleation rate.^31^ When left to evaporate naturally, the solution generated many small, orange crystals of varying sizes instead of a uniform film, as shown in Fig. 5b. To achieve a continuous film, the solution must be brought to a state of high supersaturation quickly. Although this can be done by changing temperature or pressure, uniformly heating the small fiber end is challenging. Therefore, we adopted a vacuum-assisted strategy to rapidly reduce pressure, providing an effective method for inducing the fast, widespread crystallization needed for thin film growth. As shown in Fig. 5c, under vacuum-assisted conditions, the solution rapidly crystallizes to form a uniform and continuous yellow film, which covers most of the fiber end facet. Fig. 5d shows the SEM image of the CsPbBr_3_ thin film prepared on the surface of the fiber end facet, revealing distinct, large, compact, and continuous perovskite grains. The EDS mapping of the corresponding area in Fig. 5e shows clear, strong signals of the characteristic elements Pb and Br. Finally, the photoluminescence spectrum of the sample was tested, showing a strong peak at around 545 nm (Fig. 5f), consistent with literature reports and demonstrating the good crystallinity of the thin film.^22^

## 3. Conclusion

We have demonstrated a method to confine precursor solutions on fiber end facets by implementing an area-selective wettability strategy, which creates hydrophobic sidewalls and a hydrophilic end. This approach enabled the successful *in situ* growth of MAPbBr_3_ perovskite crystals when combined with a space confinement technique using a hydrophobic glass cover. A comparison of crystal growth under natural evaporation and space confinement was conducted by combining a simplified evaporation kinetics model with the LaMer model of crystallization. This analysis clarified the distinct growth behaviors observed in different solvent systems. The analysis revealed that slow evaporation, facilitated by high-boiling-point solvents under space confinement, is the decisive factor in successfully growing perovskite crystals from the small-volume droplets held on the fiber end facet. In a complementary approach, the reliable *in situ* growth of all-inorganic CsPbBr_3_ polycrystalline thin films was achieved by employing a vacuum-assisted rapid crystallization method. The high quality of these films was confirmed by corresponding material characterizations, showing dense, continuous grain structures and strong photoluminescence. In summary, this work reports a fully controllable and versatile method for the *in situ* growth of either perovskite crystals or polycrystalline thin films on the end facets of optical fibers. This paves the way for a new generation of multifunctional ‘all-in-fiber’ optoelectronic platforms. Specifically, perovskite-integrated optical fibers are ideal for high-sensitivity, self-powered photodetectors and high-resolution imaging bundles due to the superior carrier transport of perovskites. Moreover, the high-quality polycrystalline films facilitate the development of low-threshold, fiber-integrated microlasers and nonlinear optical modulators. Furthermore, this *in situ* growth approach allows for the creation of point-of-care optical biosensors and remote radiation detectors capable of operating in confined or hazardous environments where traditional planar devices are impractical.

## 4. Experimental section

### 4.1 Materials

Methylammonium bromide (MABr) was purchased from Greatcell Solar Materials. Lead bromide (PbBr_2_) was purchased from Tokyo Chemical Industry. Cesium bromide (CsBr), poly(methyl 2-methylpropenoate) (PMMA) and trichloro(1*H*,1*H*,2*H*,2*H*-perfluorooctyl)silane (PFOCTS), dimethylformamide (DMF), dimethyl sulfoxide (DMSO) and toluene were purchased from Sigma-Aldrich. All materials were used as received without further purification.

### 4.2 Solutions preparation

The MAPbBr_3_ precursor solution was prepared by dissolving equimolar amounts of MABr and PbBr_2_ powders in a solvent system consisting of DMF, DMSO, or their mixtures inside a glass vial. The volume ratios of DMF to DMSO investigated were 0 : 5, 1 : 4, 2 : 3, 3 : 2, 4 : 1, and 5 : 0. A range of solution concentrations from 0.3 M to 1.5 M was prepared for the study. The CsPbBr_3_ precursor solution was prepared by dissolving CsBr and PbBr_2_ in a 1 : 1 molar ratio in 1 mL of DMSO to achieve a final concentration of 0.2 M. The PMMA solution was prepared by dissolving 30 mg of PMMA in a 1 : 1 volume ratio of toluene and DMF. All solutions were ultrasonically vibrated to ensure complete dissolution and were subsequently filtered through a 0.2 µm PTFE membrane filter. All solution preparation and handling steps were performed inside a nitrogen-filled glove box.

### 4.3 Area-selective wettability treatment of optical fibers

Optical fibers were stripped of their acrylic outer layer using fiber stripping pliers and then cleaved with a diamond fiber cleaver to obtain a flat end facet. The fibers were subsequently cleaned by sequential immersion and sonication in acetone and isopropanol for 10 minutes each. To render the entire silica surface hydrophilic, the fibers were treated with UV-ozone or oxygen plasma. Next, the PMMA solution was spin-coated onto a hydrophilic glass slide at 1000 rpm for 5 s. While the resulting film was still wet, an optical fiber was held vertically with its end facet oriented downwards and was brought into contact with the PMMA solution on the glass. Afterward, the fiber optic sample was placed in a glass desiccator connected to a vacuum pump. Ten microliters of PFOCTS were introduced into the chamber, which was then evacuated for 1 hour to perform a vapor-phase hydrophobic treatment. Finally, the optical fibers, now possessing hydrophobic side walls, were soaked in toluene for several minutes to completely remove the PMMA protective layer from their end surfaces. All processing procedures were performed at room temperature under atmospheric conditions.

### 4.4 *In situ* growth of perovskites on fiber end facet

All operations were monitored using a stereomicroscope equipped with a homemade optical fiber mounting stage. For MAPbBr_3_ crystal growth, a glass slide that had undergone hydrophobic treatment with PFOCTS was used for confinement. The fiber was fixed in the mount with its end facet facing vertically upwards. A small droplet of the precursor solution was transferred to the fiber end facet using a pipette tip. Immediately thereafter, a displacement platform holding the downward-facing hydrophobic glass slide was positioned over the fiber and slowly lowered until the glass made contact with the droplet. The sample was then left undisturbed, and crystallization occurred naturally through solvent evaporation in ambient conditions. The vertical gap between the optical fiber end facet and the upper hydrophobic glass slide was maintained constant throughout the crystallization process. While the gap distance was not fixed *via* high-precision mechanical stages, it was precisely regulated using a stereomicroscope to achieve an optimal precursor droplet morphology. Specifically, the upper slide was lowered until it made incipient contact with the droplet, inducing slight spreading into a stable, confined liquid meniscus conformed to the fiber facet. This empirical optimization ensures a balance between avoiding excessive constriction that would expel the precursor solution beyond the hydrophilic facet and preventing an under-confined ‘spherical cap’ geometry that would lead to excessive evaporation. Consequently, this confined volume serves as a physical template that suppresses uncontrolled vertical expansion, promoting uniform crystal thickness and high crystallinity. For CsPbBr_3_ polycrystalline thin films, the fiber was placed vertically upwards inside a vacuum drying oven. The CsPbBr_3_ solution was dropped onto the fiber end, and the chamber was evacuated to 0.02 MPa for a minimum of 20 minutes to induce rapid crystallization. All processing procedures were performed at room temperature under atmospheric conditions.

### 4.5 Material characterizations

Scanning electron microscopy (SEM) imaging and energy-dispersive X-ray spectroscopy (EDS) analysis were performed using a Hitachi Regulus 8230 Ultra-High-Resolution SEM. Photoluminescence (PL) spectra were acquired using a custom-built optical platform. The system included a PHAROS PH2 femtosecond laser operating at an excitation wavelength of 257 nm and a Horiba iHR320 spectrometer for photoluminescence detection.

## Conflicts of interest

The authors declare no conflict of interest.

## Supplementary Material

RA-016-D5RA07875J-s001

## Data Availability

All data supporting this article have been included in the main text and the supplementary information (SI). Supplementary information: a discussion of simplified droplet evaporation dynamics and figures of the wettability of different substrate conditions and different solution concentrations, as well as PL spectra of bare optical fibers. See DOI: https://doi.org/10.1039/d5ra07875j.
