# Supplementary material for: In situ growth of halide perovskite crystals and thin films on optical fiber end facets
Source: RSC Adv. 2026 Jan 19;16(5):3995–4003. doi: 10.1039/d5ra07875j (PMC12813760; doi:10.1039/d5ra07875j)
Supplement: RA-016-D5RA07875J-s001 [file RA-016-D5RA07875J-s001.pdf]

Supplementary Information (SI) for RSC Advances.
This journal is © The Royal Society of Chemistry 2026

Supplementary Information

In-Situ Growth of Halide Perovskite Single Crystals and Thin Films on Optical Fiber End Facets

Yang Yu[1], Kanak Kanti Bhowmik[1], Ruan Li[1], Kexin Li[1], Lin Zhu[1], Hai Xiao[1], Lianfeng Zhao[1,*]

[1]Holcombe Department of Electrical and Computer Engineering, Clemson University, Clemson, South Carolina 29634, United States

[*]Email: Lianfez@clemson.edu

Note 1: Geometric Model of Solvent Evaporation Kinetics

According to classical nucleation theory[1], the nucleation rate (J), number of nuclei per unit time per volume is defined as

$$J = Kexp(-\frac{\Delta G^*}{kT}) \tag{1}$$

Where K is the prefactor related to molecular dynamics, k is the Boltzmann constant and T is the absolute temperature. For a certain solution system under a given temperature, the J is determined by the critical nucleation barrier (ΔG^*), which is the change of Gibbs free energy of the system when the crystal nucleus reaches the critical nucleation radius. The ΔG^* is derived as

$$\Delta G^* = \frac{16\pi\gamma^3 v^2}{3k^2T^2(lnS)^2} \tag{2}$$

Where γ is the interfacial energy between the solution and solute molecules, v is the volume of the molecule, and S is the supersaturation of the solution. Therefore, in a specific solution system and temperature, the nucleation rate of the solution is only determined by the degree of supersaturation. For a solution that precipitates crystals by solvent evaporation with concentration increasing, the rate of solvent evaporation determines the nucleation rate. To understand why the space confinement strategy on the fiber end facet can reliably achieve single crystal growth, it is necessary to introduce geometric models to compare it with naturally evaporating droplets.

The droplet with a radius of r in the shape of a spherical cap completely covers the end facet of the

optical fiber and spreads out to form an approximately circular disk shape when covered with hydrophobic glass. The thickness of the disk is h. Therefore, its volume is $\pi r^2 h$ and the area of sidewall is $2\pi rh$. The solvent only evaporates outward on the sidewall in contact with air, and the volume of the solution decreases with time, geometrically manifested as a decrease in the contraction radius of the disk. Thus, its volume change per unit time is

$$dV = 2\pi rhdr \tag{3}$$

And the volume change per unit time is

$$\frac{dV}{dt} = -2\pi rhE \tag{4}$$

Where E is the evaporation coefficient of the solvent. Plug dV in it we obtain

$$dr = -Edt \tag{5}$$

Integrating it can yield

$$r(t) = r_0 - Et \tag{6}$$

Where r_0 is the initial radius of the droplet, in fact, it is the radius of the optical fiber. Furthermore, we can plug it in to obtain the concentration formula

$$c = \frac{n}{V} = \frac{n}{\pi r(t)^2 h} = \frac{n}{\pi (r_0 - Et)^2 h} \tag{7}$$

Where n is the amount of substance in the droplet. The volume of liquid droplets that can be bound by the fiber end facet is constant for solutions of the same concentration and solvent, determined by the wettability (contact angle) of the end face surface. In terms of time, before the crystal grows out, the solution can be considered essentially homogeneous, so it makes sense to assume that n remains constant.

For naturally evaporated droplet without confinement, its shape is a spherical cap with a body radius of R, a base radius of r and height of h', which are determined by the contact angle of the fiber end facet

$$R = \frac{r}{sin\theta} \tag{8}$$

$$h' = R(1 - cos\theta) \tag{9}$$

Due to the absence of hydrophobic glass, the solution would adhere to the hydrophilic end surface. As the solvent evaporates through the interface of air on the surface of the ball cap, the height of the ball cap will decrease with a constant radius r_0. Based on this assumption, the geometric variable under this condition is the angle of liquid ball cap and the end face chamfer, which would gradually decrease as solvent evaporating. However, the volume of the ball cap is $\pi h'^2(3R - h')/3$, and it will be very difficult to calculate the numerical value when R and h' is substituted by θ. Our goal is to make a qualitative comparison, and herein we have made an approximation based on the actual situation. Considering the initial angle, the contact angle of the end facet should be less than 10°, and it would continue to decrease as the liquid evaporates. Therefore, these geometric variables at small angles can be approximated as

$$R \approx \frac{r_0}{\theta} \tag{10}$$

$$h' \approx r_0 tan\theta \approx r_0\theta \tag{11}$$

Therefore, the exposed area of the droplet is

$$A' = 2\pi R h' \approx 2\pi {r_0}^2 \tag{12}$$

And the volume of the droplet (h << R) is

$$V' \approx \pi h'^2 R \approx \pi\theta {r_0}^3 \tag{13}$$

By substituting them into the evaporation formula, we can obtain

$$\frac{dV'}{dt} = \frac{3\pi {r_0}^3 d\theta}{dt} = -2\pi {r_0}^2 E \tag{14}$$

Cancelling $\pi {r_0}^2$ yields

$$d\theta = -\frac{2E}{3r_0} dt \tag{15}$$

Integrating the formula yields

$$\theta = \theta_0 - \frac{2E}{3r_0} t \tag{16}$$

Where θ_0 is the contact angle of the fiber end surface. Thus, the concentration is

$$c' = \frac{n}{V'} = \frac{n}{\pi\theta {r_0}^3} = \frac{n}{\pi(\theta_0 - \frac{2E}{3r_0}t){r_0}^3} \tag{17}$$

Since we have equations (7) and (17), by making the denominators equal to zero, we can obtain the termination time when the solution completely evaporates. For space confinement growth

$$t = \frac{r_0}{E} \tag{18}$$

For naturally evaporated droplet

$$t' = \frac{3\theta_0 r_0}{2E} = \frac{3\theta_0}{2}t \tag{19}$$

Because $\theta_0 < 10° \approx 0.1745$, t should larger than about $4t'$. It means that for a certain volume of solution, the evaporation time of the solvent in the spatial confinement method is at least four times that of natural exposure evaporation. For a more realistic scenario, the contact angle of completely hydrophilic silica is approximately 5 °, so this difference is even greater. The formula (17) has a steeper shape with a greater slope in the area near the asymptote. Therefore, for a determined initial concentration, the rate of concentration increase in the solution under spatial confinement is very slow, resulting in a low nucleation rate. If the evaporation coefficient of the solvent is introduced, the curve of DMSO, a high boiling point solvent, will be very smooth in the early stage, that support the growth of single crystal eventually. These simplified geometric models reliably validate the theoretical trend of controllable growth.

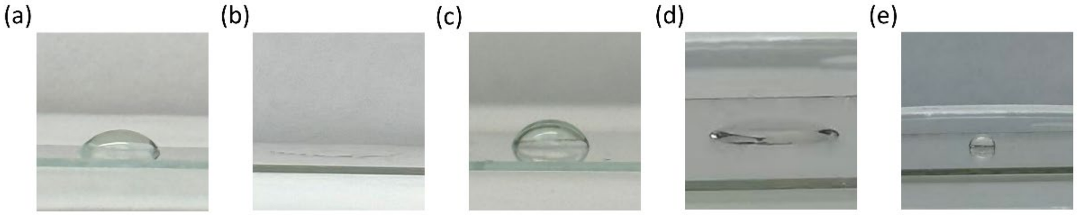

Figure S1. Side-view photographs of droplets on (a) the bare glass, (b) the glass treated by UVO, (c) the glass covered by PMMA, (d) the glass in which the PMMA was removed, and (e) the glass applied with hydrophobic treatment, respectively.

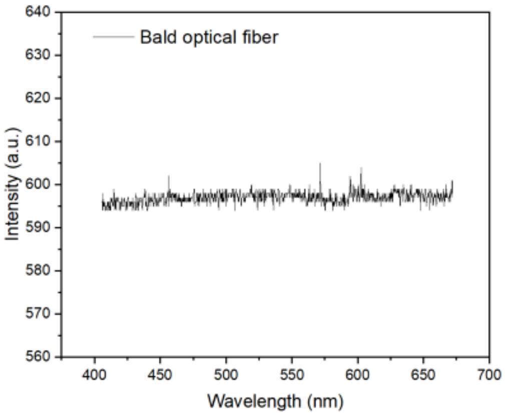

Figure S2. Photoluminescence spectrum of a bare optical fiber.

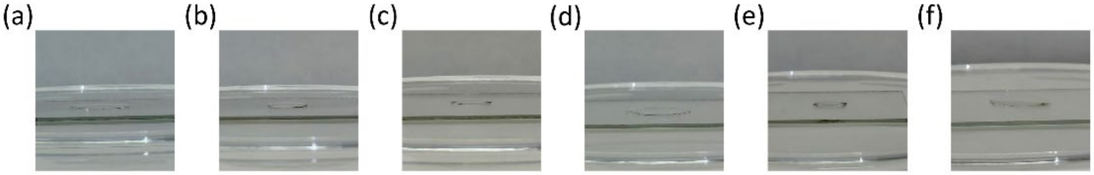

Figure S3. Side-view photographs of droplets of different perovskite precursor solutions on hydrophilic glass: (a) Pure DMF, (b) DMF:DMSO = 4:1, (c) DMF:DMSO = 3:2, (d) DMF:DMSO = 2:3, (e) DMF:DMSO = 1:4, and (f) pure DMSO, respectively.

Reference

1 *Org Process Res Dev*, 2002, 6, 201–202.
